# COVID-19 Stress and the Health of Black Americans in the Rural South

**DOI:** 10.1177/21677026211049379

**Published:** 2022-11

**Authors:** Olutosin Adesogan, Justin A. Lavner, Sierra E. Carter, Steven R. H. Beach

**Affiliations:** 1 Department of Psychology, University of Georgia; 2 Department of Psychology, Georgia State University; 3 Center for Family Research, University of Georgia

**Keywords:** Black Americans, COVID-19, mental health, physical health, social determinants of health

## Abstract

Black Americans have been disproportionately affected by the COVID-19 pandemic. To better understand changes in and predictors of their mental and physical health, in the current study, we used three waves of data (two prepandemic and a third during summer 2020) from 329 Black men and women in the rural South. Results indicated that health worsened after the onset of the pandemic, including increased depressive symptoms and sleep problems and decreased self-reported general health. Greater exposure to COVID-19-related stressors was significantly associated with poorer health. Prepandemic stressors (financial strain, racial discrimination, chronic stress) and prepandemic resources (marital quality, general support from family and friends) were significantly associated with exposure to COVID-19-related stressors and with health during the pandemic. Findings underscore how the pandemic posed the greatest threats to Black Americans with more prepandemic psychosocial risks and highlight the need for multifaceted interventions that address current and historical stressors among this population.

The ongoing Coronavirus Disease 2019 (COVID-19) pandemic has upended life in the United States and globally. Beyond the staggering number of infections, hospitalizations, and deaths due to COVID, the pandemic has been a significant source of stress for most individuals and families (e.g., American Psychological Association, 2020), with harmful consequences for physical and mental health. For example, in a February 2021 survey led by the American Psychological Association (2021), 61% of adults reported experiencing undesired weight changes since the start of the pandemic, and 67% reported sleeping more or less than they wanted during this period. Mental-health difficulties have been elevated as well (e.g., Xiong et al., 2020). Centers for Disease Control and Prevention (CDC) data indicate that the prevalence of anxiety and depressive disorder symptoms in summer 2020 were approximately 3 to 4 times higher than the previous year (Czeisler et al., 2020). Longitudinal studies reveal similar within-persons changes. One national study found that the rate of significant mental distress increased 50% from 2018 and 2019 to April 2020 (Pierce et al., 2020).

Although the pandemic has devastated all demographic groups, its impact has been unevenly distributed; Black Americans and members of other racial and ethnic minority groups have been disproportionately affected. To better understand the psychological and physical health of Black Americans during the COVID-19 pandemic, in the current study, we used data from an ongoing longitudinal study of Black families in the rural South. Studying the impact of COVID-19 among Black Americans in this region is particularly important because Southern states were among the last to shut down and the first to reopen and had fewer pandemic-related restrictions relative to other areas of the United States. Moreover, the rural South is a region in which systemic racism and oppression have resulted in elevated poverty rates for Black Americans (e.g., Burton et al., 2017), and health disparities have been well documented (National Academies of Sciences, Engineering, and Medicine, 2017), including during the COVID-19 pandemic (Rushovich et al., 2021). In the current study, we aim to provide new insights regarding changes in self-reported psychological and physical health from before to during the pandemic among this sample, how COVID-19-related stressors are associated with their functioning, and how their prepandemic stressors (financial strain, racial discrimination, chronic stress) and resources (marital quality, support from family and friends) predict exposure to COVID-19-related stress and functioning.

## The Impact of the COVID-19 Pandemic on Black Americans

In the early weeks of the pandemic, a number of scholars warned about its likely harms for Black Americans given initial data revealing race-based disparities in morbidity and mortality (e.g., Dyer, 2020; Millett et al., 2020; Yancy, 2020) and other risk factors resulting from structural racism, such as chronic health conditions, unemployment, lack of insurance, medical mistreatment, overrepresentation in essential service industries, and environmental risk (Egede & Walker, 2020; Poteat et al., 2020). More than a year later, race-based disparities in COVID morbidity and mortality in the United States remain. As of March 2021, Black Americans were at 1.1 times greater risk for COVID-19 infection, 2.9 times greater risk for COVID-19 hospitalization, and 1.9 times greater risk for death due to COVID-19 relative to White Americans (CDC, 2021a). In Georgia, where the current study takes place, COVID-19 mortality rates among Black men and women (128.5 deaths/100,000 and 84.1 deaths/100,000, respectively) have been substantially higher than among White men and women (53.2 deaths/100,000 and 38.2 deaths/100,000, respectively; Rushovich et al., 2021). These trends likely contribute to Black Americans being more likely than members of other racial and ethnic groups to know someone who has been hospitalized or died as a result of having COVID-19 and being more likely than other groups to view COVID-19 as a major threat to the health of the U.S. population and to their personal health (Johnson & Funk, 2021).

Black Americans have been disproportionately affected by the COVID-19 pandemic in other ways as well, including the degree to which they experienced pandemic-related stressors and psychological and physical-health disturbances. In spring 2020, Black Americans were more likely than White Americans to experience a job or wage loss because of the pandemic (44% and 38%, respectively; Lopez et al., 2020) and experienced higher levels of food insufficiency, rent or mortgage defaults, and inaccessible medical care relative to other racial and ethnic groups as well (Chakrabarti et al., 2021). In the February 2021 survey by the American Psychological Association mentioned earlier, rates of unintended physical changes were elevated among Black Americans: 64% reported undesired changes to weight, and 76% reported undesired changes to sleep (American Psychological Association, 2021). Black Americans were also more likely than other groups to report concerns about the future; 54% reported that they did not feel comfortable going back to living life as they had before the pandemic (vs. 44% of White participants), and 57% reported feeling uneasy about adjusting to in-person interactions after the pandemic ends (vs. 47% of White participants; American Psychological Association, 2021). There were disparities in mental-health difficulties as well. CDC data indicated that Black Americans reported elevated rates of anxiety or depressive symptoms over the past 7 days in both August 2020 and January 2021 (37.7% and 44.5%, respectively) relative to White Americans during these periods (35.4% and 39.8%, respectively; Vahratian et al., 2021). Worryingly, at both time points, Black Americans were also less likely than White Americans to be taking prescription medication for mental health or receiving therapy or counseling during the previous 4 weeks (15.6% vs. 25.6% in August 2020 and 18.7% vs. 28.1% in January 2021; Vahratian et al., 2021). These patterns, combined with preexisting disparities because of structural racism, make Black Americans particularly vulnerable to mental-health difficulties because of the COVID-19 pandemic (e.g., Novacek et al., 2020).

## Predicting Black Americans’ Mental and Physical Health During the COVID-19 Pandemic

Given the challenges characterizing the experiences of Black Americans during the COVID-19 pandemic, it is important to identify risk and protective factors that predict their mental and physical health during this period. McCubbin and Patterson’s (1983a, 1983b) double ABCX model of family stress and adaptation following a crisis provides a useful framework for conceptualizing these issues (for an application of this model to Black families during the COVID-19 pandemic, see Chaney, 2020). This model outlines how families’ postcrisis adaptation is partly a function of the “pileup of demands” that arise because of the crisis. In the case of the COVID-19 pandemic, we would therefore expect that greater exposure to COVID-19-related stressors (e.g., having difficulty getting essentials, working outside the home, losing income) should be associated with worse outcomes for Black Americans. Examining how exposure to COVID-19-related stressors is associated with psychological and physical health during the pandemic is particularly important because, as noted earlier, Black Americans as a group were especially likely to experience pandemic-related stressors (e.g., job or wage loss, food insecurity, inaccessible medical care; Chakrabarti et al., 2021; Lopez et al., 2020).

Note that the double ABCX model argues that stressors and resources that are in place before the crisis affect this pileup process. This incorporation of preexisting factors makes this model particularly valuable for understanding the experiences of Black Americans during the COVID-19 pandemic. Well before the pandemic, Black families commonly faced financial strain because of structural racism (e.g., Burton et al., 2017), interpersonal racial discrimination (e.g., Lee et al., 2019; D. R. Williams, 2018), and overall chronic stress (from employment, health, parenting, etc.). Acknowledging the existence of these stressors and considering how they may be associated with subsequent outcomes is thus crucial. On the basis of the double ABCX model, we would expect that these preexisting stressors would predict exposure to COVID-19-related stressors (i.e., the postcrisis pileup of demands) and predict mental and physical health during the pandemic (i.e., postcrisis adaptation). The double ABCX model also highlights the importance of precrisis resources. Among Black Americans, relationships with romantic partners, extended family, and friends are highly valued (e.g., Gaines et al., 1997) and have been identified as important sources of resilience as individuals adapt to stress (e.g., Chaney, 2020; Lincoln & Chae, 2010; Odom et al., 2010). Accordingly, as with preexisting stressors, we would expect there might also be linkages between preexisting resources such as romantic relationship quality and social support from family and friends and exposure to COVID-19-related stressors and mental and physical health during the pandemic. In this manner, the double ABCX model suggests that Black Americans’ functioning during the pandemic could reflect both their degree of exposure to COVID-19-related stressors and their preexisting psychosocial characteristics.

## The Current Study

To address these possibilities and better understand the psychological and physical health of Black Americans during the COVID-19 pandemic, in the current study, we used data from an ongoing longitudinal study of Black families in the rural South. The sample was assessed five times before the pandemic and once during the summer of 2020 (June 2020–September 2020), which was several months into the pandemic and a period when COVID-19 infections and deaths in Georgia increased steadily (Georgia Department of Public Health, n.d.). Our longitudinal design uniquely positions us to address several research questions about participants’ experiences:

*Research Question 1:* How does self-reported health change from before the COVID-19 pandemic to during the pandemic?

For this descriptive aim, we considered multiple indices of health for which we had previous data available, including self-reported depressive symptoms, sleep problems, and general health. Our analysis of within-persons changes builds on previous work showing that adults’ self-reported physical and mental health has worsened because of the COVID-19 pandemic (e.g., American Psychological Association, 2021; Czeisler et al., 2020; Pierce et al., 2020). We focused these analyses on changes from the most recent assessment preceding the COVID-19 pandemic (Wave 5) to during the pandemic (Wave 5.5). Enhancing our analyses, we also examined depressive symptoms and sleep problems from a second, earlier prepandemic wave of data collection (Wave 4) and compared these symptoms with later functioning. Doing so allowed us to test the possibility that any observed recent changes could simply reflect more general trends. We predicted that, consistent with previous findings, self-reports of health will worsen from prepandemic to during the pandemic, whereas reports from the two prepandemic waves will not differ.

We then turned to examining several questions that draw on the double ABCX model (McCubbin & Patterson, 1983a, 1983b) to test how postcrisis adaptation is affected by the pileup of demands and precrisis factors.

*Research Question 2:* How is level of exposure to COVID-19-related stressors associated with participants’ self-reported health during the pandemic?

Specifically, we tested whether the degree to which Black American individuals experienced COVID-related stressors (e.g., job loss, the diagnosis of a family member) was associated with their self-reported depressive symptoms, sleep problems, general health, overall daily impact of the pandemic, and perceived stress due to the pandemic. We predicted that greater exposure to COVID-19-related stressors would be associated with worse self-reported mental and physical health during the pandemic, consistent with the double ABCX model’s assertion that the pileup of demands following a crisis is associated with adaptation.

*Research Question 3:* How are prepandemic stressors associated with exposure to COVID-19-related stressors and with health during the pandemic?

**Fig. 1.**
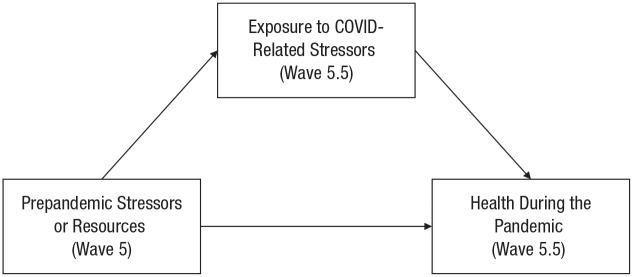
General analytic model showing direct and indirect effects of prepandemic stressors or resources on health during the pandemic through exposure to COVID-19-related stressors.

We considered three separate indices of prepandemic stress: (a) financial strain, given the elevated poverty rates in the rural South as a result of systemic racism and oppression (e.g., Burton et al., 2017); (b) racial discrimination, given that this is a chronic and ubiquitous stressor affecting Black Americans (e.g., Lee et al., 2019; D. R. Williams, 2018); and (c) a general index of chronic stress (e.g., employment, health, parenting). We tested the hypothesis that the COVID-19 pandemic exacerbated existing structural disparities such that individuals with more stressors before the pandemic subsequently experienced more COVID-19-related stressors and poorer health. We also drew on the double ABCX model to test indirect-effect models whereby prepandemic stressors influenced health during the pandemic through level of exposure to COVID-19-related stressors (Fig. 1).

*Research Question 4:* How are prepandemic resources associated with level of exposure to COVID-19-related stressors and health during the pandemic?

We assessed two social resources: the quality of the couple relationship and general support from family and friends. As with the previous aim, we examined bivariate associations between these domains, COVID-19-related stressors, and health during the pandemic and also tested indirect-effect models whereby prepandemic resources influenced health during the pandemic through level of exposure to COVID-19-related stressors (Fig. 1).

For all research questions, we report separate results for women and men given evidence (e.g., Alon et al., 2020; Keeter, 2021) that they may be experiencing the pandemic differently.

## Method

### Participants

In this study, we used data from the Protecting Strong African American Families (ProSAAF) project, a randomized controlled trial of a family-centered intervention to promote strong couple, coparent, and parent–child relationships in two-parent African American families (for a full study overview, see Barton et al., 2018).^1^ Couples with an African American child between the ages of 9 and 14 took part in the study.^2^ All participants lived in small towns and communities in the southern United States, where poverty rates are among the highest in the nation and unemployment rates are above the national average (DeNavas-Walt & Proctor, 2014). To be eligible, couples (a) had to have been in a relationship for 2 years or more, (b) had to be living together, and (c) had to have been coparenting an African American child in the targeted age range for at least 1 year. Couples had to be willing to spend 6 weeks engaged in a family-centered prevention program and not be planning to move out of the study area during that period. Families were recruited by mail and phone via advertisements distributed in their communities and through lists that local schools provided. Schools in 16 counties provided information on youths in Grades 4 through 6.

Subject enrollment began in 2013 and continued into 2014. After recruitment, staff visited the families’ homes to explain the study in further detail and obtain consent. Six hundred ninety-two individuals (from 346 families) participated in the baseline assessment in 2013–2014. Nearly all participants were involved in mixed-sex relationships (*n* = 688 individuals from 344 families; 99.4%); four individuals from two families were headed by a female same-sex couple.^3^ Families completed four additional waves of in-home assessments before the onset of the COVID-19 pandemic. In the current study, we used data from the two most recent waves before the pandemic (Wave 4, collected between 2015 and 2016, when 569 individuals participated, and Wave 5, collected between 2018 and March 2020, when 366 individuals participated^4^) and a third wave collected by telephone during the COVID-19 pandemic (Wave 5.5, collected between June 2020 and September 2020, when 329 individuals participated). Each adult was compensated with a $50 check for participating in the Wave 5.5 assessment. All procedures were approved by the University of Georgia Institutional Review Board (study title: “Protecting Strong African American Families”).

Given our focus on COVID-19-related functioning, only the 329 participants who completed the Wave 5.5 assessment were included in the current study (55% of baseline participants; 90% of the eligible Wave 5 participants). The sample consisted of 191 (58%) Black women (mean age = 43.02 years, *SD* = 8.12) and 138 (42%) men (mean age = 47.36 years, *SD* = 10.24). Many of the participants participated with their partner as well: There were 130 families in which both the female and the male partner participated (*n* = 260 individuals), 61 families in which only the female partner participated (*n* = 61 individuals), and eight families in which only the male partner participated (*n* = 8 individuals). At the time of the Wave 5.5 assessment, 68% of women and 61% of men were employed; 17% of women and 6% of men were teleworking, and 87% of women and 95% of men were going to their workplaces in person (participants could endorse more than one option). In the most recent reports of family income (at Wave 5), median annual income was $20,000 to $24,000 (range = < $10,000 to $150,000–$199,999).

### Measures

#### Exposure to COVID-19-related stressors

Individuals’ exposure to COVID-19-related stressors during the pandemic was assessed at Wave 5.5 using the COVID-19 Exposure and Family Impact Survey (CEFIS; Kazak et al., 2020). This survey included 22 items assessing specific challenges that families may have had to face because of the COVID-19 pandemic (sample items: “A member of the family lost their job permanently” and “Someone in the family was hospitalized for COVID-19”). Responses were 0 = no and 1 = yes. Items on this measure were summed to create a total score, and higher scores indicate greater exposure to COVID-related stressors.

#### Health outcomes

##### Depressive symptoms

Depressive symptoms were assessed at Wave 4, Wave 5, and Wave 5.5 using a 10-item, short-form of the Center for Epidemiologic Studies Depression Scale (CES-D; Kohout et al., 1993; Radloff, 1977; sample item: “How often did you feel depressed?” in the past week). Response options ranged from 0 (*rarely or none of the time [0–1 days]*) to 3 (*most or all of the time [6–7 days]*). The total score was calculated by summing the items; higher scores indicate more depressive symptoms (women: αs = .75 at Wave 4, .64 at Wave 5, and .69 at Wave 5.5; men: αs = .55 at Wave 4, .42 at Wave 5, and .78 at Wave 5.5). This scale has previously demonstrated good reliability and validity in Black American populations (Canady et al., 2009; Makambi et al., 2009; C. D. Williams et al., 2007).

##### General health

General health was assessed at Wave 5 and Wave 5.5 using a two-item questionnaire adapted from the Medical Outcomes Study (MOS) 36-item short-form health survey (McHorney et al., 1994; Ware & Sherbourne, 1992). Previous research across diverse ethnic groups and patient populations has found that individuals completing measures of self-reported health provide valuable information regarding their own health status (Jylhä, 2009; Larsson et al., 2002; Nielsen & Krasnik, 2010). These measures can provide insight into the process by which contextual life stress can lead to poor physical-health and mental-health outcomes and mortality. The Spearman-Brown (SB) coefficient was calculated for this scale because this statistic is less biased than either Cronbach’s α or the Pearson correlation for evaluating the reliability of two-item measures (Eisinga et al., 2013). The first item on this scale is “In general, would you say your health is?” Responses on this item ranged from 1 (*excellent*) to 5 (*poor*). The second item on this scale was “Compared to one year ago, how would you rate your health in general now?” Responses on this item ranged from 1 (*much better now than 1 year ago*) to 5 (*much worse than 1 year ago*). Both items on this scale were reverse-scored and then summed to create a total score, and higher scores indicate better general health (SB women: *r*s = .62 at Wave 5 and .57 at Wave 5.5; SB men: *r*s = .61 at Wave 5 and .45 at Wave 5.5).

##### Sleep problems

Sleep problems were assessed at Wave 4, Wave 5, and Wave 5.5 using a six-item sleep scale adapted from the MOS Sleep Scale (Spritzer & Hays, 2003; sample item: “How often did you awaken from your sleep and have trouble falling asleep again?”). Responses on these items ranged from 1 (*none of the time*) to 5 (*all of the time*), and higher scores indicate more sleep problems (women: αs = .78 at Wave 4, .73 at Wave 5, and .75 at Wave 5.5; men: αs = .63 at Wave 4, .59 at Wave 5, and .76 at Wave 5.5).

##### Perceived impact and stress due to COVID-19

Individuals’ perceptions of impact and stress due to the pandemic were assessed at Wave 5.5 using two items from the COPE Coronavirus Perinatal Experiences Impact Survey (Thomason et al., 2020). To assess impact, participants were asked, “What has been the overall level of daily impact due to the COVID-19 outbreak?” Item responses ranged from 1 (*no impact*) to 7 (*extreme impacts*). To assess stress due to the pandemic, participants were asked, “What has been your overall level of stress related to the COVID-19 outbreak?” Item responses ranged from 1 (*no stress*) to 7 (*extreme stress*).

#### Prepandemic stressors

##### Financial strain

Women and men’s levels of prepandemic financial strain were assessed at Wave 5 using a composite of individual ratings from Conger et al.’s (1994) Financial Adjustment and Unmet Material Needs scales. The Financial Adjustment scale included 11 items assessing the specific needs that families had to forgo because of financial hardship during the past 12 months (sample item: “Has your family reduced or eliminated medical insurance because of financial need?”). Responses were 0 = no and 1 = yes. Items on this measure were summed to create a total score, and higher scores reflect more financial adjustments (women: α = .86; men: α = .80). The Unmet Material Needs scale included four items assessing participants’ perception of their family’s ability to meet their needs (sample item: “My family has enough money to afford the kind of home we need”). The response set ranged from 1 (*strongly disagree*) to 4 *(strongly agree*). For this measure, each item was reverse-scored; all items were summed to create a total score, and higher scores indicate more unmet need (women: α = .84; men: α = .81).

Scores on the two scales were highly correlated (*r*s = .47–.50; all *p*s < .05). Accordingly, we created a composite score by first creating standard scores for each scale and then summing the standard scores. Higher scores on the composite indicate more financial strain.

##### Racial discrimination

Prepandemic experiences of racial stigma and racial stress were assessed for women and men at Wave 5 using the Racism and Life Experiences Scale (Harrell, 2000). This measure includes nine items assessing personal experiences of racial stigma (sample item: “Have you been overlooked, ignored, or not given service because of your race?” in the past 6 months). Responses for these items ranged from 1 (*never*) to 4 (*frequently*). Items were summed, and higher scores indicate more racial discrimination (women: α = .85; men: α = .92).

##### Chronic stress

To assess prepandemic levels of chronic stress, women and men completed a questionnaire adapted from the UCLA Life Stress Interview (Hammen et al., 1987). Participants were provided a list of 13 domains (e.g., employment, parenting, family relationships, health) and asked to rate how stressful they found each domain at Wave 5. Responses for items on this scale ranged from 0 (*not at all stressful*) to 2 (*extremely stressful*). Items were summed to create a total score in which higher scores indicate higher levels of chronic stress (women: α = .84; men: α = .80).

#### Prepandemic resources

##### Marital quality

Prepandemic marital quality was assessed at Wave 5 using the six-item Quality of Marriage questionnaire (Norton, 1983). This widely used questionnaire includes five items assessing aspects of an individual’s relationship (sample item: “My partner and I have a good relationship”). Responses on these items ranged from 1 (*strongly disagree*) to 5 (*strongly agree*). The questionnaire also includes one item stating, “Which best describes the degree of happiness, everything considered, in your relationship?” Responses on this item ranged from 1 (*very unhappy*) to 5 (*perfectly happy*). Items were summed to create a total score, and a higher total score indicates greater marital quality (women: α = .95; men: α = .93).

##### General support from family and friends

Prepandemic general support from family and friends was assessed at Wave 5 using a six-item scale (Cohen & Hoberman, 1983). This measure includes items assessing the quality and nature of individuals’ relationships with friends and family members (sample item: “How much do your friends make you feel appreciated, loved, or cared for? Is it . . . ”). Item responses ranged from 1 (*a lot*) to 3 (*not at all*). Items were reverse-coded and summed such that a higher score indicates greater support from friends and family (women: α = .85; men: α = .78).

Descriptive statistics and bivariate correlations for all study variables are presented in Table 1.

**Table 1.** Descriptive Statistics and Correlations Among Study Variables

Variable	Correlation											Women			Men		
	1	2	3	4	5	6	7	8	9	10	11	*N*	*M*	*SD*	*N*	*M*	*SD*
1. COVID-19-related stressors (W5.5)	—	.22**	.19*	.21*	−.30**	−.21*	.25**	−.18*	.32**	.24**	.30**	191	6.81	3.12	138	6.29	3.19
2. Financial strain (W5)	.16*	—	.16	.35**	−.19*	−.20*	−.02	−.15	.11	.10	.17*	189	0.00	1.71	138	−0.02	1.71
3. Racial discrimination (W5)	.24**	.21**	—	.23**	−.03	−.03	.08	−.11	.25**	.13	.19*	190	18.64	5.67	138	16.70	6.09
4. Chronic stress (W5)	.16*	.55**	.19**	—	−.25**	−.17*	.13	−.14	.24**	.09	.13	190	6.32	4.19	138	5.32	4.61
5. Marital quality (W5)	−.29**	−.20*	−.20*	−.19*	—	.20*	−.05	.03	−.09	−.20*	−.22*	129	24.22	5.89	121	25.93	4.72
6. General support from family and friends (W5)	−.02	−.26**	.01	−.34**	.09	—	−.21*	.18*	−.17	−.05	−.18*	181	12.04	5.60	138	15.50	2.15
7. Depression (W5.5)	.40**	.15*	.14	.22**	−.41**	−.01	—	−.28**	.52**	.05	.27**	188	9.28	5.74	138	6.86	5.17
8. General health (W5.5)	−.20**	−.23**	−.08	−.29**	.17	.06	−.26**	—	−.34**	−.03	−.22*	190	5.86	1.66	136	5.77	1.53
9. Sleep problems (W5.5)	.43**	.18*	.23**	.26**	−.28**	.03	.60**	−.34**	—	.22*	.32**	190	14.92	4.36	137	12.89	4.02
10. Overall daily impact from COVID-19 (W5.5)	.28**	.07	.16*	.11	−.19*	.02	.31**	−.26**	.31**	—	.68**	191	5.37	1.59	138	5.07	1.85
11. Overall stress due to COVID-19 (W5.5)	.21**	−.08	.15*	.08	−.18*	.08	.36**	−.31**	.37**	.55**	—	191	5.25	1.65	138	4.72	1.90

Note: The *r* values for women are below the diagonal, and the *r* values for men are above the diagonal. W5 was before the pandemic; W5.5 was after the onset of the pandemic. These findings address Research Questions 2 through 4. W5 = Wave 5; W5.5 = Wave 5.5.

* *p* < .05 (two-tailed). **p < .01 (two-tailed).

### Analytic plan

Analyses were conducted using IBM SPSS (Version 26.0) and the PROCESS Macro (Hayes, 2017). To test Research Question 1, we conducted a series of paired sample *t* tests to examine changes in health after the onset of the COVID-19 pandemic. To test Research Question 2, we used correlational analyses to examine bivariate associations between exposure to COVID-19-related stressors and health during the pandemic. To test Research Question 3, we first used bivariate correlations to examine associations between prepandemic stressors and exposure to COVID-19-related stressors and between prepandemic stressors and health during the pandemic. We then used longitudinal mediation models (Fig. 1) to examine the direct and indirect effects of prepandemic stressors on health through exposure to COVID-19-related stressors; indirect effect analyses have been recommended as a rigorous method to test mediation (e.g., Rucker et al., 2011). We tested these models using the PROCESS macro in IBM SPSS (Hayes, 2017) using 5,000 bootstrap estimates to generate 95% confidence intervals. Predictor variables were mean centered in each model. To test Research Question 4, we repeated the steps from Research Question 3 to examine associations between prepandemic resources, exposure to COVID-19-related stressors, and health during the pandemic. We conducted all analyses separately for women and men given the nonindependence of the data (because participants were recruited from families).

## Results

### Research Question 1: changes in health after the onset of the COVID-19 pandemic

We began by examining changes in participants’ mental and physical health from prepandemic (Waves 4 and 5) to during the pandemic (Wave 5.5). Results indicated that participants’ health significantly worsened after the onset of the pandemic along multiple dimensions (see Table 2). For women, average levels of depression significantly increased from Wave 4 to Wave 5.5, change in (Δ) *M* = 1.78, *p* < .001, and from Wave 5 to Wave 5.5, Δ*M* = 2.56, *p* < .001. In contrast, average levels of depression significantly decreased across the two prepandemic assessments (Wave 4 to Wave 5, Δ*M* = −0.75, *p* = .04). In addition, women’s sleep problems significantly increased after the onset of the pandemic. Specifically, sleep problems significantly increased from Wave 4 to Wave 5.5, Δ*M* = 0.95, *p* = .01, and from Wave 5 to Wave 5.5, Δ*M* = 1.01, *p* < .001. There were no significant changes in sleep problems across the two prepandemic assessments (Wave 4 to Wave 5 Δ*M* = −0.09, *p* > .05). Finally, women’s general health significantly decreased from Wave 5 to Wave 5.5, Δ*M* = −0.47, *p* < .001; general health was not assessed at Wave 4.

**Table 2.** Paired Samples *t* Tests Comparing Health Before and After the Onset of the COVID-19 Pandemic

Outcomes	Women						Men					
	*N*	Δ*M*	*SD*	*t*	*df*	*p*	*N*	Δ*M*	*SD*	*t*	*df*	*p*
Wave 4 to Wave 5												
Depression	188	−0.75	4.87	−2.10	187	**.04**	133	0.35	4.28	0.93	132	.35
Sleep problems	188	−0.09	5.13	−0.24	187	.81	133	0.41	3.61	1.30	132	.19
Wave 5 to Wave 5.5												
Depression	187	2.56	5.41	6.50	186	**.00**	138	0.71	5.59	1.49	137	.14
Sleep problems	189	1.01	4.72	2.96	188	**.00**	137	−0.40	4.19	−1.12	136	.27
General health	189	−0.47	1.79	−3.61	188	**.00**	136	−0.39	1.69	−2.69	135	**.01**
Wave 4 to Wave 5.5												
Depression	186	1.78	5.85	4.16	185	**.00**	133	0.99	5.42	2.11	132	**.04**
Sleep problems	188	0.95	4.86	2.68	187	**.01**	132	−0.03	4.27	−0.08	131	.94

Note: Boldface type indicates significant effects. Δ = change. Waves 4 and 5 were before the pandemic; Wave 5.5 was after the onset of the pandemic. General health was not assessed at Wave 4, so changes from Wave 4 to Wave 5 and from Wave 4 to Wave 5.5 are not reported. These findings address Research Question 1.

For men, average levels of depression significantly increased from Wave 4 to Wave 5.5, Δ*M* = 0.99, *p* = .04, but did not significantly differ from Wave 5 to Wave 5.5, Δ*M* = 0.71, *p* > .05. There were no significant changes in men’s depression across the prepandemic assessments (Wave 4 and Wave 5, Δ*M* = 0.35, *p* > .05). There were also no significant changes in men’s sleep problems across any of the waves (all *p*s > .05), although men’s ratings of general health significantly decreased from Wave 5 to Wave 5.5, Δ*M* = −0.39, *p* = .01.

### Research Question 2: associations between exposure to COVID-related stressors and health

Correlations were used to examine associations between exposure to COVID-19-related stressors and mental and physical health during the pandemic (i.e., depressive symptoms, sleep problems, general health, perceived impact of the pandemic on daily life, and perceived stress due to the pandemic). Results, shown in Table 1, indicated that exposure to COVID-19-related stressors was associated with poorer health. Specifically, for both women and men, exposure to COVID-19-related stressors was significantly positively associated with depressive symptoms (women: *r* = .40; men *r* = .25) and sleep problems (women: *r* = .43; men: *r* = .32) and significantly negatively associated with general health (women: *r* = −.20; men: *r* = −.18). In addition, exposure to COVID-19-related stressors was significantly positively correlated with perceived impact of the pandemic on daily life (women: *r* = .28; men: *r* = .24) and perceived level of stress due to the pandemic (women: *r* = .21; men: *r* = .30).

### Research Question 3: prepandemic stressors, exposure to COVID-related stressors, and health

#### Bivariate associations

Next, we examined correlations between prepandemic stressors and (a) exposure to COVID-related stressors and (b) health during the pandemic (Table 1). These results indicated that prepandemic stressors were significantly positively associated with exposure to COVID-19-related stressors such that individuals experiencing more stress before the pandemic reported experiencing more COVID-related stressors. Specifically, for both women and men, prepandemic financial strain (women: *r* = .16; men: *r* = .22), racial discrimination (women: *r* = .24; men: *r* = .19), and chronic stress (women: *r* = .16; men: *r* = .21) at Wave 5 were significantly associated with exposure to COVID-19-related stressors at Wave 5.5.

Experiences of prepandemic stress were also significantly associated with health during the pandemic. For women, prepandemic financial strain and chronic stress were both significantly positively associated with levels of depression (financial strain: *r* = .15; chronic stress: *r* = .22) and sleep problems (financial strain: *r* = .18; chronic stress: *r* = .26) during the pandemic. Women’s prepandemic chronic stress was also significantly negatively associated with their general health during the pandemic (*r* = −.29). Women’s experiences of prepandemic racial discrimination were significantly positively associated with their sleep problems during the pandemic (*r* = .23), level of daily impact due to the pandemic (*r* = .16), and stress due to the pandemic (*r* = .15). For men, prepandemic experiences of racial discrimination (*r* = .25) and chronic stress (*r* = .24) were both significantly positively associated with sleep problems. In addition, men’s financial strain (*r* = .17) and racial discrimination (*r* = .19) were both significantly positively associated with their perceived stress due to the pandemic.

#### Indirect-effect models

We ran a series of indirect-effect models to test whether prepandemic stressors predicted health during the pandemic through elevated exposure to COVID-19-related stressors (see Table 3). For women, there were significant indirect effects from prepandemic financial strain and from prepandemic chronic stress to depression, sleep problems, perceived daily impact of the pandemic, and perceived stress due to the pandemic (but not general health) through exposure to COVID-19-related stressors. There were also significant indirect effects from prepandemic racial discrimination to all five outcomes through exposure to COVID-19-related stressors. There remained a few significant direct effects as well, including from prepandemic financial strain to general health, prepandemic racial discrimination to sleep problems, and prepandemic chronic stress to depression, general health, and sleep problems.

**Table 3.** Direct and Indirect Effects of Prepandemic Stressors on Health During the Pandemic Through Exposure to COVID-19-Related Stressors

Prepandemic stressor (W5) and health during the pandemic (W5.5)	*N*	Indirect effect through exposure to COVID-19-related stressors (*a* × *b*)			Direct effect (*c*′)		
		Coefficient	Boot *SE*	95% CI	Coefficient	*SE*	*p*
Results for women							
Financial strain							
Depression	186	**0.20**	**0.10**	**[0.020, 0.400]**	0.29	0.23	.21
General health	188	−0.02	0.02	[−0.062, 0.001]	**−0.20**	**0.07**	**.01**
Sleep problems	188	**0.16**	**0.08**	**[0.017, 0.338]**	0.30	0.17	.08
Perceived daily impact of COVID-19	189	**0.04**	**0.02**	**[0.003, 0.090]**	0.02	0.07	.74
Perceived stress due to COVID-19	189	**0.04**	**0.02**	**[0.003, 0.084]**	−0.11	0.07	.12
Racial discrimination							
Depression	187	**0.09**	**0.03**	**[0.037, 0.162]**	0.05	0.07	.52
General health	189	**−0.01**	**0.01**	**[−0.028, −0.002]**	−0.01	0.02	.68
Sleep problems	189	**0.07**	**0.03**	**[0.029, 0.125]**	**0.11**	**0.05**	**.04**
Perceived daily impact of COVID-19	190	**0.01**	**0.01**	**[0.002, 0.029]**	0.03	0.02	.16
Perceived stress due to COVID-19	190	**0.01**	**0.01**	**[0.002, 0.030]**	0.03	0.02	.16
Chronic stress							
Depression	187	**0.09**	**0.04**	**[0.026, 0.171]**	**0.21**	**0.09**	**.03**
General health	189	−0.01	0.01	[−0.027, 0.001]	**−0.11**	**0.03**	**.00**
Sleep problems	189	**0.08**	**0.03**	**[0.020, 0.140]**	**0.20**	**0.07**	**.00**
Perceived daily impact of COVID-19	190	**0.02**	**0.01**	**[0.002, 0.037]**	0.03	0.03	.33
Perceived stress due to COVID-19	190	**0.01**	**0.01**	**[0.001, 0.030]**	0.02	0.03	.53
Results for men							
Financial strain							
Depression	138	**0.18**	**0.10**	**[0.026, 0.400]**	−0.24	0.26	.35
General health	136	**−0.03**	**0.02**	**[−0.080, −0.001]**	−0.10	0.08	.19
Sleep problems	137	**0.15**	**0.08**	**[0.031, 0.332]**	0.12	0.20	.56
Perceived daily impact of COVID-19	138	**0.06**	**0.04**	**[0.006, 0.140]**	0.06	0.09	.55
Perceived stress due to COVID-19	138	**0.07**	**0.04**	**[0.014, 0.157]**	0.12	0.09	.19
Racial discrimination							
Depression	138	**0.04**	**0.02**	**[0.004, 0.096]**	0.03	0.07	.65
General health	136	**−0.01**	**0.01**	**[−0.021, 0.000]**	−0.02	0.02	.36
Sleep problems	137	**0.03**	**0.02**	**[0.005, 0.082]**	**0.13**	**0.05**	**.02**
Perceived daily impact of COVID-19	138	**0.01**	**0.01**	**[0.001, 0.033]**	0.03	0.03	.33
Perceived stress due to COVID-19	138	**0.02**	**0.01**	**[0.003, 0.036]**	0.04	0.03	.11
Chronic stress							
Depression	138	**0.05**	**0.03**	**[0.006, 0.134]**	0.09	0.10	.34
General health	136	−0.01	0.01	[−0.027, 0.000]	−0.04	0.03	.22
Sleep problems	137	**0.05**	**0.03**	**[0.006, 0.110]**	**0.16**	**0.07**	**.02**
Perceived daily impact of COVID-19	138	**0.02**	**0.01**	**[0.002, 0.050]**	0.02	0.03	.60
Perceived stress due to COVID-19	138	**0.02**	**0.01**	**[0.004, 0.054]**	0.03	0.03	.37

Note: Boldface type indicates significant effects. These findings address Research Question 3. CI = confidence interval; W5 = Wave 5; W5.5 = Wave 5.5.

For men, there were significant indirect effects from all three prepandemic stressors (i.e., financial strain, racial discrimination, chronic stress) to all five health outcomes through exposure to COVID-19-related stressors, with the exception of chronic stress to general health. There were also two significant direct effects for men: from prepandemic racial discrimination to sleep problems and from prepandemic chronic stress to sleep problems.

### Research Question 4: prepandemic resources, exposure to COVID-related stressors, and health

#### Bivariate associations

As with prepandemic stressors, we first used correlations to examine associations between prepandemic resources and (a) exposure to COVID-19-related stressors and (b) health during the pandemic (see Table 1). Prepandemic resources were significantly associated with exposure to COVID-related stressors and health during the pandemic. Specifically, for women and men, marital quality was significantly negatively associated with later exposure to COVID-19-related stressors (women: *r* = −.29; men: *r* = −.30). For men only, general support from family and friends was also significantly associated with later exposure to COVID-19-related stressors (*r* = −.21).

Prepandemic resources were also significantly associated with health during the pandemic. For women, marital quality was significantly negatively associated with depression (*r* = −.41), sleep problems (*r* = −.28), perceived daily impact of the COVID-19 pandemic (*r* = −.19), and perceived stress due to the COVID-19 pandemic (*r* = −.18). For men, marital quality was significantly negatively associated with perceived daily impact of the pandemic (*r* = −.20) and perceived stress due to the pandemic (*r* = −.22) but not with any of the other health variables (all *p* > .05). In addition, for men, general support from friends and family was significantly negatively associated with depression (*r* = −.21) and perceived stress due to the pandemic (*r* = −.18) and significantly positively associated with general health (*r* = .18).

#### Indirect-effect models

Finally, we ran a series of indirect-effect models to test whether prepandemic resources predicted health during the pandemic through elevated exposure to COVID-19-related stressors (see Table 4). For women, there were significant indirect effects from marital quality to depression and sleep problems through exposure to COVID-19-related stressors. There was also a significant direct effect of women’s marital quality on their depression. For men, there were significant indirect effects from both sources of support (i.e., marital quality, general support from friends and family) to depression, sleep problems, and perceived stress due to the pandemic through exposure to COVID-19-related stressors. There was also a significant indirect effect from men’s marital quality to their general health and a significant indirect effect from general support to perceived daily impact of the pandemic. Finally, there was a significant direct effect of men’s general support from friends and family on depression.

**Table 4.** Direct and Indirect Effects of Prepandemic Resources on Health During the Pandemic Through Exposure to COVID-19-Related Stressors

Prepandemic resource (W5) and health during the pandemic (W5.5)	*N*	Indirect effect through exposure to COVID-19-related stressors (*a* × *b*)			Direct effect (*c*′)		
		Coefficient	Boot *SE*	95% CI	Coefficient	*SE*	*p*
Results for women							
Marital quality							
Depression	128	**−0.10**	**0.04**	**[−0.179, −0.031]**	**−0.31**	**0.08**	**.00**
General health	129	0.01	0.01	[−0.003, 0.030]	0.03	0.02	.16
Sleep problems	129	**−0.10**	**0.03**	**[−0.167, −0.035]**	−0.11	0.06	.06
Perceived daily impact of COVID-19	129	−0.01	0.01	[−0.031, 0.008]	−0.04	0.02	.09
Perceived stress due to COVID-19	129	−0.01	0.01	[−0.038, 0.001]	−0.03	0.02	.16
General support from family and friends							
Depression	178	−0.01	0.03	[−0.066, 0.062]	−0.01	0.07	.91
General health	180	0.00	0.01	[−0.010, 0.012]	0.02	0.02	.41
Sleep problems	180	−0.01	0.03	[−0.058, 0.047]	0.03	0.05	.60
Perceived daily impact of COVID-19	181	0.00	0.01	[−0.017, 0.014]	0.01	0.02	.64
Perceived stress due to COVID-19	181	0.00	0.01	[−0.013, 0.011]	0.03	0.02	.22
Results for men							
Marital quality							
Depression	121	**−0.09**	**0.05**	**[−0.198, −0.018]**	0.04	0.10	.72
General health	119	**0.02**	**0.01**	**[0.002, −0.044]**	−0.01	0.03	.76
Sleep problems	120	**−0.08**	**0.03**	**[−0.154, −0.023]**	0.00	0.08	.98
Perceived daily impact of COVID-19	121	−0.02	0.02	[−0.057, 0.002]	−0.05	0.04	.14
Perceived stress due to COVID-19	121	**−0.03**	**0.02**	**[−0.070, −0.008]**	−0.06	0.04	.13
General support from family and friends							
Depression	138	**−0.11**	**0.07**	**[−0.267, −0.008]**	**−0.40**	**0.20**	**.05**
General health	136	0.02	0.02	[0.000, 0.062]	0.11	0.06	.09
Sleep problems	137	**−0.11**	**0.06**	**[−0.247, −0.016]**	−0.20	0.16	.21
Perceived daily impact of COVID-19	138	**−0.04**	**0.03**	**[−0.105, −0.004]**	0.00	0.07	.97
Perceived stress due to COVID-19	138	**−0.05**	**0.03**	**[−0.114, −0.009]**	−0.11	0.07	.15

Note: Boldface type indicates significant effects. These findings address Research Question 4. CI = confidence interval; W5 = Wave 5; W5.5 = Wave 5.5.

## Discussion

In the current study, we used longitudinal data from an ongoing study of Black families living in the rural South to assess changes in adults’ self-reported mental and physical health after the onset of the COVID-19 pandemic, examine how exposure to COVID-related stress was associated with their health during the pandemic, and test associations between prepandemic stressors and resources, subsequent exposure to COVID-19-related stressors, and health during the pandemic. It remains important to understand mental and physical health among this population given that morbidity and mortality from COVID-19 have been disproportionately high among Black Americans (CDC, 2021a). Furthermore, Black Americans have experienced worse mental and physical health during the pandemic relative to White Americans but were simultaneously less likely to receive professional help for mental-health difficulties and had more difficulty accessing medical care (e.g., American Psychological Association, 2021; Chakrabarti et al., 2021; Vahratian et al., 2021). Black Americans have also faced heightened strains during the pandemic, such as financial insecurity and employment uncertainty (e.g., Chakrabarti et al., 2021; Lopez et al., 2020), and were made uniquely vulnerable during this period because of historical and modern inequities from structural racism (Novacek et al., 2020; Poteat et al., 2020).

### Summary of findings

Addressing our first research question, results indicated that participants’ health worsened after the onset of the COVID-19 pandemic. For both Black men and women, levels of depression increased, and self-reported general health decreased. In addition, women experienced an increase in sleep problems after the onset of the pandemic. These results build on earlier findings indicating that mental health during the pandemic is notably worse than in preceding years (e.g., Czeisler et al., 2020) and that many adults report physical-health difficulties as well (American Psychological Association, 2021). These results also add to longitudinal studies revealing within-persons changes in mental distress from prepandemic to early pandemic (e.g., Pierce et al., 2020). Note that these changes are not simply a reflection of longer trends given that there was no evidence for similar changes in a comparison of the two prepandemic waves. Accordingly, we can more strongly conclude that these changes reflect—at least in part—consequences of the COVID-19 pandemic.

Enhancing our confidence in this interpretation, we found evidence that health during the COVID-19 pandemic was significantly associated with participants’ level of exposure to COVID-19-related stressors, and that there were worse outcomes among individuals who reported experiencing more stressors (Research Question 2). These outcomes include higher levels of depression and sleep problems, greater perceived daily impact and overall stress due to the COVID-19 pandemic, and lower levels of self-reported general health. Combined with the first set of findings, these findings highlight the consequential impact of acute stress resulting from the COVID-19 pandemic in two ways: first, by showing how generally, Black individuals’ health worsened after the onset of the pandemic; and second, by showing how experiencing more stressors related to the COVID-19 pandemic was associated with poorer health. The latter finding is consistent with McCubbin and Patterson’s (1983a, 1983b) double ABCX model of family stress and adaptation following a crisis by indicating that the extent of pileup of stressors and demands during a crisis affects adaptation during and after the crisis. These findings also underscore meaningful variability in Black individuals during the pandemic, and the worst outcomes were evident among those individuals who experienced the most COVID-related stressors.

Strikingly, our findings indicated that COVID-related stressors and mental-health and physical-health outcomes were associated with prepandemic stressors as well (Research Question 3). We found that prepandemic financial strain, experiences of racial discrimination, and chronic stress were all significantly positively correlated with subsequent exposure to COVID-related stressors. In addition, prepandemic financial strain, racial discrimination, and chronic stress were associated with poorer health outcomes during the pandemic. Indirect-effect analyses indicated that for both women and men, there were significant associations between prepandemic stressors and health during the pandemic through exposure to COVID-19-related stress. These findings provide further evidence for the double ABCX model by indicating that stressors before a crisis (i.e., the COVID-19 pandemic) contribute to a pileup of stressors and demands during the crisis and, thus, lead to poorer outcomes and adaptation during and after the crisis. The findings also highlight the adverse effects of specific prepandemic contextual stressors that disproportionately affect Black lives, such as racial discrimination and financial strain. In the current sociopolitical climate, the current findings begin to consider the toll that a pileup of chronic stressors rooted in oppression takes and the ways in which that toll is further exacerbated by the pandemic. More generally, these findings show that even in the face of a universal stressor such as the COVID-19 pandemic, the degree of impact may be most pronounced among people who were already experiencing high levels of stress beforehand.

Finally, addressing our fourth research question, we found that prepandemic resources appeared to be a promising resilience factor associated with positive outcomes in the face of the COVID-19 pandemic. Specifically, for both Black American women and men, prepandemic marital quality was significantly negatively associated with later exposure to COVID-19-related stressors. In addition, for women, marital quality was also significantly associated with lower levels of depression, sleep problems, perceived stress, perceived daily impact due to the pandemic, and higher levels of general health. For men, marital quality was significantly negatively associated with perceived stress and impact due to the pandemic. There were also significant indirect effects for women and men from prepandemic marital quality to multiple dimensions of mental and physical health during the pandemic through exposure to COVID-19-related stressors, consistent with the double ABCX model. These findings add to previous work that has highlighted the importance of romantic relationships for Black Americans in the face of stress (e.g., Beach et al., 2019; Lincoln & Chae, 2010).

Note that general support from family and friends was a significant promotive factor only for men. For men, general support from family and friends was significantly negatively associated with depression and stress due to the pandemic and had significant indirect effects on multiple health outcomes through greater exposure to COVID-19-related stressors. For women, general support from family and friends was not significantly associated with any health outcome, and there were no significant direct or indirect effects of general support on their health. Although it is important not to overinterpret these effects given that we did not test for gender differences, it is possible that they could reflect men’s greater orientation to larger social networks (Baumeister & Sommer, 1997). We note, however, that previous research has found associations between social support and psychological functioning among rural Black women (e.g., Odom et al., 2010; Seawell et al., 2014), which suggests that this remains an important resource among this group as well. Additional research is needed to further understand these patterns and to examine other resources that may affect functioning in the face of COVID-19 and other extreme stressors.

Taken together, these findings add to the existing literature on health effects during the COVID-19 pandemic among Black Americans and expand this literature through the use of longitudinal data to highlight changes in health from before to after the onset of the pandemic and how pandemic impacts are associated with prepandemic stressors and social resources. In so doing, these findings show the potential for compounding stress effects on individuals and families already experiencing high levels of stress (e.g., financial strain or racial discrimination) or low levels of protective resources (e.g., marital quality or general social support) before the pandemic. More broadly, these patterns support arguments raised by a number of scholars that preexisting social disparities and vulnerabilities, including those driven by structural racism, increase risk for poor mental-health and physical-health outcomes among Black Americans during the COVID-19 pandemic (e.g., Egede & Walker, 2020; Novacek et al., 2020; Poteat et al., 2020). We note that results were generally consistent across women and men despite other evidence that they may be experiencing the pandemic differently (e.g., Alon et al., 2020; Keeter, 2021), which suggests that these patterns characterize participants’ lives irrespective of gender.

### Strengths and limitations

The study had several notable strengths, including its focus on Black Americans living in the rural South, a population intensely and disproportionately affected by the onset of the pandemic. In addition, our use of longitudinal data that included prepandemic assessments allowed us to assess changes in functioning over time and to examine how preexisting stressors and resources were associated with subsequent COVID-19-related stress and functioning during the pandemic. Another strength was that our measures of preexisting stressors (particularly financial strain and racial discrimination) and COVID-19-related stressors both centered on discrete experiences (e.g., having enough money to meet needs, COVID hospitalizations in the family) rather than subjective perceptions of “feeling stressed,” which reduces concerns that the significant associations across these domains simply reflect dispositional traits.

There are also limitations that are important to acknowledge. First, because the study focused exclusively on Black Americans in the rural South, the extent to which these findings generalize to Black Americans living in other regions is unclear. Second, all health and functioning outcomes were assessed through self-report. Future research would benefit from the use of more objective measures of health (e.g., actigraphy data for sleep) in conjunction with self-report measures to create a more complete picture of individuals’ functioning. Third, a few of the measures had relatively low reliabilities, and some constructs were assessed with one or two items. Although this is not uncommon for broad survey assessments, more rigorous and reliable assessments would allow for stronger conclusions. Fourth, we conducted a large number of statistical analyses, which could increase risk for Type I error. We note, however, that we have focused our discussion on overall patterns, which were generally consistent across specific analyses. Fifth, we analyzed data from men and women separately. Although this allowed us to better understand gender-specific patterns in light of findings indicating that women and men may be experiencing the pandemic differently (e.g., Alon et al., 2020; Keeter, 2021), analyzing data in a multilevel model collapsing across gender would have allowed us to examine sample-level effects and resulted in greater power to detect small effects.

Finally, because assessments took place during summer 2020, these findings focus on functioning during the first months of the pandemic and cannot speak to functioning during the fall and winter months. Given that the toll of the pandemic increased during this period (CDC 2021b), it is possible that these patterns might be even more pronounced had we assessed participants during this period. Indeed, data from the CDC reveal that the percentage of Black adults reporting symptoms of depression or an anxiety disorder over the past 7 days increased from 37.7% in late August 2020 to 44.5% in January 2021 (Vahratian et al., 2021). In addition, this study period overlapped with the weeks and months following the murder of George Floyd, a period in which the killing, frequent media coverage, and public debate resulted in racial retraumatization for many Black Americans and increases in their depressive and anxious symptoms (Fowers & Wan, 2020). It is thus possible that some of the changes in self-reported physical and mental health we observed when examining Research Question 1 in this sample could have been the result of vicarious discrimination and/or trauma related to this and subsequent events in addition to stress from the COVID-19 pandemic. Greater attention to the intersecting impact of these dual pandemics on Black Americans’ mental and physical health is needed.

### Future directions and implications

Findings from this study raise a number of additional questions for future research. First, given that local government policies regarding social distancing, masks, mandatory lockdowns, and other precautions varied a great deal from region to region in the United States, future research could examine whether the patterns of stress effects shown in this study vary in regions with different policies (e.g., Chakrabarti et al., 2021). Second, our study highlighted the potential benefits of social relationships. Future research could further explore whether these and other resources also exert stress-buffering effects that counter the impact of preexisting and concurrent stress. Third, the U.S. government enacted a number of interventions to stabilize individuals and families and minimize harms during the pandemic, including additional payments to families through stimulus checks, enhanced unemployment benefits, and restrictions on evictions. It will be important to study whether Black Americans will be at increased risk for poor mental, physical, and social outcomes if these policies are no longer in place, particularly given evidence that Black Americans were at elevated risk for food insufficiency and rent or mortgage defaults (Chakrabarti et al., 2021). Finally, as the toll of the pandemic becomes clearer, it will be important to examine how the disproportionate death rate among Black Americans affects the mental and physical health among their surviving family members and friends. Even before the COVID-19 pandemic, Black Americans were significantly more likely than White Americans to experience the death of a family member throughout their life span, including the loss of a parent or sibling as a child and the loss of a spouse or child as an adult (Umberson et al., 2017). The losses from the COVID-19 pandemic will likely amplify these patterns and compound the effects of stress, bereavement, and cumulative disadvantage on Black Americans’ health.

The results of this study also have important practical implications. In particular, these findings highlight the need for multifaceted interventions for Black Americans that address both the acute stress caused by the COVID-19 pandemic and more chronic, preexisting contextual stressors, such as racial discrimination and financial stress. Individual-level and systems-level interventions will both be necessary to alleviate stress and promote positive health and functioning for Black individuals living in the rural South and Black Americans more broadly. Individual interventions that capitalize on already established sources of support and resilience, such as social support and romantic relationships, may also be beneficial for improving individual mental-health and physical-health outcomes. In addition, researchers have foregrounded the importance of systems-level interventions that work to counteract centuries of structural racism and inequality that have led to health inequities among Black Americans. For example, Egede and Walker (2020) provided a number of suggestions for systems-level approaches to intervention that could be implemented during and after the COVID-19 pandemic, including education for medical professionals about the history of racism in medicine, community engagement, policies aimed at economic empowerment, and community-level support for enhancing neighborhood stability. Both individual- and systems-level interventions will be necessary to counteract poor outcomes highlighted in this study that have been caused both by the COVID-19 pandemic and by preexisting chronic life stressors due to structural inequities in society.

### Conclusion

In conclusion, the present study contributes to a growing body of literature showing the harms of the COVID-19 pandemic for the health of Black Americans. Our findings indicate that the onset of the pandemic was associated with negative changes in physical and mental health among Black adults living in the rural South, and poorer functioning was more pronounced among people experiencing higher levels of exposure to pandemic-related stress and with greater prepandemic psychosocial risks (more stressors, weaker social resources). These findings underscore how the ongoing COVID-19 crisis has been most damaging to people who are most vulnerable and call for multifaceted interventions that address historical and current disparities to promote resilience during and beyond the pandemic.

## Transparency

*Action Editor:* Darby Saxbe

*Editor:* Jennifer Tackett

*Author Contributions*

S. R. H. Beach secured funding and oversaw data collection. O. Adesogan, J. A. Lavner, and S. R. H. Beach developed the study concept. All of the authors contributed to the study design. O. Adesogan performed the data analysis and interpretation under the supervision of J. A. Lavner. O. Adesogan and J. A. Lavner wrote the initial manuscript, and S. E. Carter and S. R. H. Beach provided critical revisions. All of the authors revised and approved the final manuscript for submission.

## Notes

1. Throughout this section, we use the term “African American” to be consistent with our original recruitment materials, but otherwise use the more general term “Black American.”

2. Eligibility guidelines required the target child to be African American but not both parents. Racial and ethnicity information was not assessed in surveys completed by participants. Notes from research staff indicated that two caregivers (from different families) were not African American. All participants were comfortable being identified as part of an African American family.

3. None of the individuals involved in a same-sex relationship participated in Wave 5.5 data collection, and thus they were not included in the current analyses.

4. An additional 29 participants completed Wave 5 data collection after a state of public-health emergency given that the pandemic was declared on March 14, 2020, and they were not approached for participation in Wave 5.5 because of overlap in assessment windows.
